# Malaria parasitaemia and mRDT diagnostic performances among symptomatic individuals in selected health care facilities across Ghana

**DOI:** 10.1186/s12889-021-10290-1

**Published:** 2021-01-28

**Authors:** Benjamin Abuaku, Linda Eva Amoah, Nana Yaw Peprah, Alexander Asamoah, Eunice Obeng Amoako, Dickson Donu, George Asumah Adu, Keziah Laurencia Malm

**Affiliations:** 1 Epidemiology Department, Noguchi Memorial Institute for Medical Research, College of Health Sciences, University of Ghana, P. O. Box LG581, Legon, Ghana; 2 Immunology Department, Noguchi Memorial Institute for Medical Research, College of Health Sciences, University of Ghana, P. O. Box LG581, Legon, Ghana; 3 National Malaria Control Programme, Public Health Division, Ghana Health Service, Accra, Ghana

**Keywords:** Malaria parasitaemia, Symptomatic individuals, Health care facilities

## Abstract

**Background:**

Parasitological diagnosis generates data to assist malaria-endemic countries determine their status within the malaria elimination continuum and also inform the deployment of proven interventions to yield maximum impact. This study determined prevalence of malaria parasitaemia and mRDT performances among febrile patients in selected health care facilities across Ghana.

**Methods:**

This study was a cross-sectional survey conducted in the previously 10 regions of Ghana from May to August 2018. Each patient suspected to have uncomplicated malaria was tested using microscopy and two malaria rapid diagnostic tests (mRDTs): routinely used CareStart™ Malaria HRP2 (Pf) and SD Bioline Malaria Ag Pf (HRP2/pLDH). Main outcome variables were malaria slide and CareStart™ Malaria HRP2 (Pf) positivity rates; and diagnostic accuracy of CareStart™ Malaria HRP2 (Pf) and SD Bioline Malaria Ag Pf (HRP2/pLDH) using microscopy as “gold standard”.

**Results:**

Overall parasite positivity rates were 32.3% (6266/19402) by mRDT and 16.0% (2984/18616) by microscopy, with *Plasmodium falciparum* mono-infection accounting for 98.0% of all infections. The odds of parasitaemia by microscopy was significantly lower among female patients compared with males (OR = 0.78; 95% CI: 0.66–0.91), and among patients with history of previous antimalarial intake compared with those with no such history (OR = 0.72; 95% CI: 0.54–0.95). Overall sensitivity of CareStart™ Malaria HRP2 (Pf) was statistically similar to that of the HRP2 band of SD Bioline Malaria Ag Pf (HRP2/pLDH) combo kit (95.4%; 95% CI: 94.6–96.1 vs 94.3%; 95% CI: 93.4–95.1; *p* = 0.065) but significantly higher than the pLDH band (89.3%; 95% CI: 88.1–90.4; *p* < 0.001). The same pattern was observed for negative predictive value.

**Conclusions:**

Malaria control interventions should be targeted at the general population, and history of antimalarial intake considered a key predictor of malaria slide negativity. Furthermore, HRP2-based mRDTs remain effective diagnostic tool in the management of suspected uncomplicated malaria in the country.

**Supplementary Information:**

The online version contains supplementary material available at 10.1186/s12889-021-10290-1.

## Background

Malaria remains one of the major public health problems in Ghana as in other sub-Saharan African countries. The WHO African region accounted for 93.0% of the 228 million global cases and 94.0% of the 405,000 global deaths that occurred in 2018 [1]. Ghana, listed as one of the 10 African countries with the highest burden of malaria, was reported as being one of the three African countries with the highest absolute increase in malaria cases in 2018 compared with 2017 [1]. Malaria accounted for 21.8% of total admissions and 1.5% of total deaths in Ghana in 2018 [2].

The objective of uncomplicated malaria case management is to cure the infection as rapidly as possible in order to prevent progression to severe disease and reduce transmission to others. Key to this is prompt and accurate parasitological diagnosis, which is also critical in assessing impact of preventive strategies such as long lasting insecticidal nets (LLINs), indoor residual spraying (IRS), larviciding/larval source management, seasonal malaria chemoprevention (SMC), and intermittent preventive treatment in pregnancy (IPTp) [3, 4]. Parasitological diagnosis among febrile patients provides data that assists malaria-endemic countries to determine their malaria status within the malaria elimination continuum [5–7]. Additionally, elucidating determinants of malaria parasitaemia is useful in informing the deployment of proven interventions to yield maximum impact [8–10]. We report the prevalence of malaria parasitaemia and mRDT diagnostic performances among febrile patients who participated in a nation-wide survey on HRP2 gene deletion conducted in selected health care facilities between May and August 2018.

## Methods

### Study sites

**Fig. 1:**
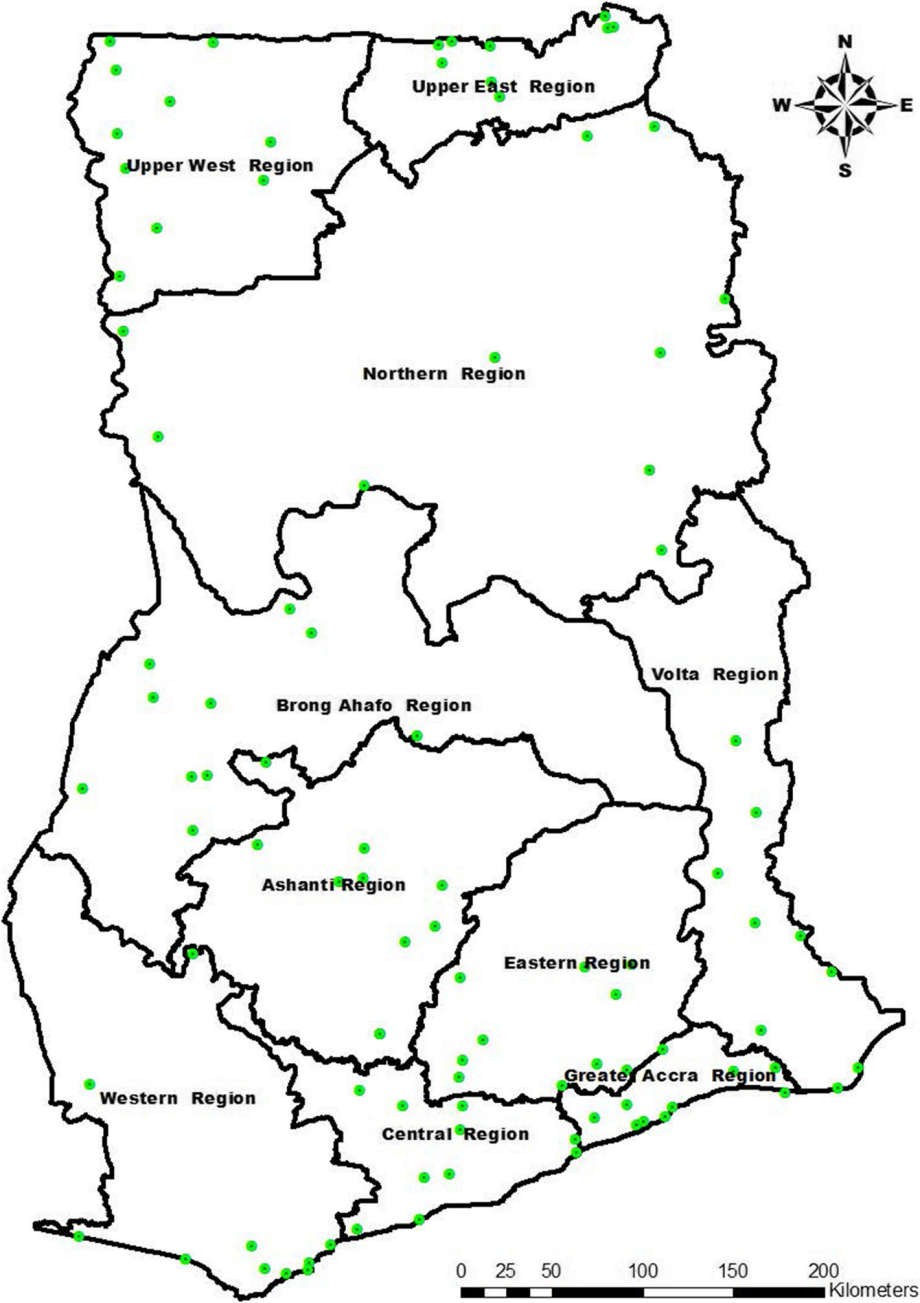
Map of Ghana showing study sites. Legend details: Green dots represent the study sites. The map was created by Mr. George Asumah Adu. We obtained shapefiles from the Centre for Remote Sensing and Geographic Information Services (CERGIS), University of Ghana, Legon, Accra, and used ArcGIS 10.3.1 to plot the GPS coordinates

A total of 10 health care facilities were selected in each of the then 10 regions of Ghana (Fig. 1) based on probability proportional to size (PPS) using average monthly OPD suspected malaria cases for year 2016 as a measure of facility size (data source: District Health Information Management System 2 - DHIMS 2). The primary sampling units in each region were therefore health care facilities within the region. The cumulative total of average monthly OPD suspected malaria cases was then used to determine the sampling interval (SI) for each region. This was followed by computer generation of random numbers between 1 and the SI to determine the Random Start (RS) for each region. Subsequent facilities in each region were selected following the series RS; RS + SI; RS + 2SI; ……..RS+(*d*-1)*SI [11], where *d* is the facility/cluster number.

A minimum sample size of 370 *Pf* positive cases, by microscopy, was generated for each region based on estimated *pf*HRP2 gene deletion prevalence of 3.2% at 95% confidence interval, 0.022 margin of error, and a design effect of 1.5 using 10 clusters per region. A total of 20,000 suspected malaria cases (2000 per region) were to be screened to get the minimum sample size of 370 based on an estimated average malaria slide positivity rate of 20.0% (unpublished data).

### Study design

The study was a cross-sectional survey among all symptomatic individuals seeking care in selected health care facilities in the previously 10 regions of Ghana during the early phase of the peak transmission season of 2018 (May – August). This was part of a study to estimate the prevalence of *pfhrp2/pfhrp3* gene deletions using the 2018 WHO protocol [12].

### Data collection

Trained Nurses used the KoBoCollect application to administer standard questionnaires to all suspected uncomplicated malaria patients visiting the outpatient department of the selected health care facilities. Prior to this, informed consent (for adult participants), parental consent (for children under 18 years), and assent (for children aged 12–17 years) were obtained for all participants. Data collected included participant’s age, gender, educational background, marital status, and antimalarial intake during the 2-week period preceding survey (Additional file 1).

### Malaria testing

Malaria rapid diagnostic test (mRDT) was performed by trained laboratory Technicians / Technologists according to manufacturer’s instructions using the standard test kit in use routinely (i.e. CareStart™ Malaria HRP2 (Pf)) as well as a stocked test kit (i.e. SD Bioline Malaria Ag Pf (HRP2/pLDH)). All patients were exposed to the two mRDTs. Thick and thin blood smears for malaria microscopy were also prepared. The thin film was fixed in methanol, and both thick and thin films stained with 3.0% Giemsa stain for approximately 45 min and air-dried. All slides were stored in plastic slide boxes for later examination by WHO-certified expert microscopists.

### Determination of malaria parasitaemia by expert microscopists

The thick film was used for parasite quantification per white blood cells (WBCs) whilst the thin film was used for species identification. As per WHO guidelines, the thin film was used for parasite quantification per parasitized red blood cells (RBCs) in cases where ≥100 parasites were observed in each thick film field under × 100 objective. Parasite quantifications per WBCs were computed per microliter (μL) of blood assuming 8000 WBCs/μL whilst counts per parasitized RBCs were computed per μL of blood assuming an average of 5,000,000 RBCs per μL [13]. A blood slide was declared negative when no parasites were seen after the examination of 200 thick film fields. For quality control (QC) purposes 10.0% of slides as well as all positive slides were examined by 2 independent expert microscopists. Initially examined positive slides that were reported by the QC microscopist as negative or with different species were re-examined by a third independent expert microscopist, and two examinations with the same results considered as final.

### Data management and statistical analyses

Two main datasets (questionnaire and microscopy) were captured separately using KoBoCollect. The datasets were extracted and cleaned separately in IBM SPSS Statistics version 21 using data quality tabulations and source data (e.g. facility laboratory record books), where necessary. The 2 datasets were subsequently merged after matching the individual IDs for analyses. Main outcome variables analyzed were malaria slide and mRDT positivity rates (only for routinely used CareStart™ Malaria HRP2 (Pf)); and mRDT (i.e. both CareStart™ Malaria HRP2 (Pf) and SD Bioline Malaria Ag Pf (HRP2/pLDH)) sensitivity (SEN) and specificity (SPEC) as well as positive predictive value (PPV) and negative predictive value (NPV) using microscopy as the “gold standard”. The following formulae were applied:

$$ \mathrm{SEN}=\mathrm{Number}\ \mathrm{of}\ \mathrm{true}\ \mathrm{positives}\ \left(\mathrm{TP}\right)/\left[\mathrm{Number}\ \mathrm{of}\ \mathrm{TP}+\mathrm{Number}\ \mathrm{of}\ \mathrm{false}\ \mathrm{negatives}\ \left(\mathrm{FN}\right)\right] $$

$$ \mathrm{SPEC}=\mathrm{Number}\ \mathrm{of}\ \mathrm{true}\ \mathrm{negatives}\ \left(\mathrm{TN}\right)/\left[\mathrm{Number}\ \mathrm{of}\ \mathrm{TN}+\mathrm{Number}\ \mathrm{of}\ \mathrm{false}\ \mathrm{positive}\ \left(\mathrm{FP}\right)\right] $$

$$ \mathrm{PPV}=\mathrm{Number}\ \mathrm{of}\ \mathrm{TP}/\left[\mathrm{Number}\ \mathrm{of}\ \mathrm{TP}+\mathrm{Number}\ \mathrm{of}\ \mathrm{FP}\right)\Big] $$

$$ \mathrm{NPV}=\mathrm{Number}\ \mathrm{of}\ \mathrm{TN}/\left[\mathrm{Number}\ \mathrm{of}\ \mathrm{TN}+\mathrm{Number}\ \mathrm{of}\ \mathrm{FN}\right)\Big] $$

Explanatory variables for malaria slide and mRDT positivity rates were participant’s age, gender, educational background, marital status, and history of antimalarial intake within the two-week period preceding survey. Analysis of educational level was controlled for patients aged < 5 years because official age for basic education in Ghana is 6 years [14]. Similarly, analysis of marital status was controlled for patients aged < 15 years because the legal age of marriage in Ghana is 18 years [15]. The < 15 years threshold also provided the opportunity to capture those who were married or cohabiting before the legal age of 18 years. Associations between parasite positivity and the explanatory variables were determined by univariate analyses (Chi-square and Fishers exact tests significant at *p* < 0.05). Variables showing significant associations were subsequently used in a multivariable logistic analysis to determine their independent effects. To account for clustering of measurements of individuals within the same health care facility and region, the Generalized Estimating Equation (GEE) approach in R was used to assess the associations between explanatory variables and malaria slide (microscopy) and mRDT positivity rates (significant at *p* < 0.05). Taking cognizance of the fact that GEE takes into account the dependency of observations by specifying a working correlation structure, four working correlation structures were identified: independence, exchangeable, auto regressive and unstructured. These parameter estimates were still valid even when the working correlation structures were mis-specified [16, 17]. Following the selection of the working assumption, the model-based and empirical parameter estimates with its standard errors for each working correlation structure was obtained. This was done to define the most appropriate working assumptions. A working assumption was considered appropriate when the standard errors of both empirical and model-based estimates were close to each other. The odds logistic was subsequently applied after obtaining estimates from the GEE.

## Results

### Background characteristics of participants

**Fig. 2:**
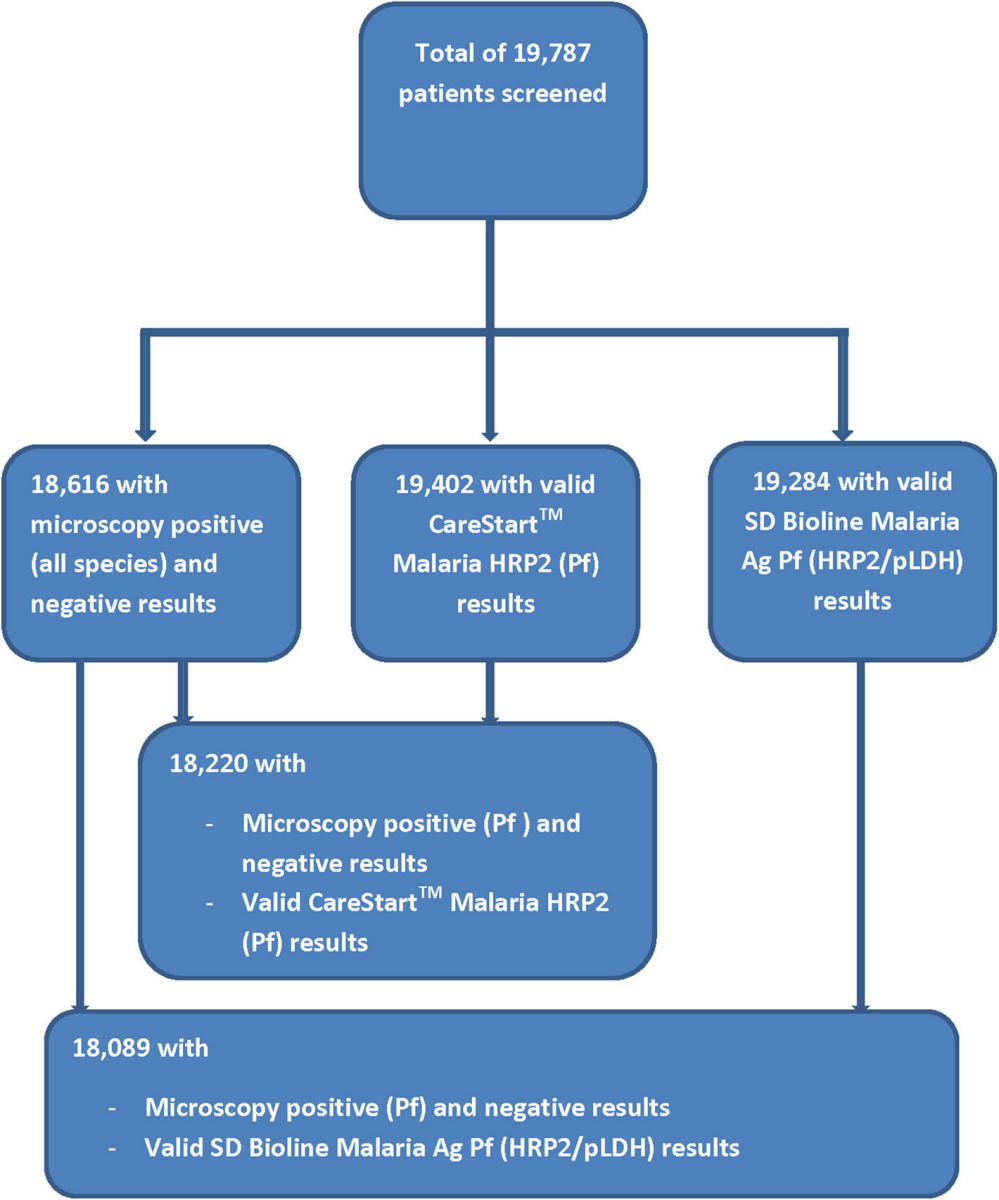
Participant flow showing number screened and number with microscopy and valid mRDT results

A total of 19,787 suspected malaria cases from the 10 regions of Ghana were screened during the study period (Fig. 2). Majority of participants were female (64.9%), aged 20 years and above (51.0%), had primary education (30.1%), married (58.6%), and had not taken any antimalarial within the 2-week period preceding the survey (90.3%) (Table 1). Valid CareStart™ Malaria HRP2 (Pf), SD Bioline Malaria Ag Pf (HRP2/pLDH) and microscopy results were available for 19,402 (98.1%), 19,284 (97.5%), and 18,616 (94.1%), respectively (Fig. 2). Examination of the 10.0% QC slides showed a discordance of 8.1% between first and second microscopists (Kappa value of 0.78).

**Table 1:** Background characteristics of study participants

Characteristics	n	%
Gender (*N* = 19,109)		
Male	6698	35.1
Female	12,411	64.9
Age group (yrs) (*N* = 19,114)		
< 5	3119	16.3
5–9	2885	15.1
10–14	1660	8.7
15–19	1708	8.9
≥ 20	9742	51.0
^a^Education (*N* = 15,498)		
None	3657	23.6
Primary	4667	30.1
Junior High	3044	19.6
Senior High	2520	16.3
Tertiary	1610	10.4
^b^Marital status (*N* = 10,953)		
Single	4000	36.5
^c^Married	6420	58.6
^d^Divorced	533	4.9
^e^PAMI (*N* = 19,787)		
No	17,871	90.3
Yes	1916	9.7

^a^Controlled for patients aged < 5 yrs

^b^Controlled for patients aged < 15 yrs

^c^Includes cohabiting

^d^Includes separated

^e^Previous antimalarial intake

### Prevalence of malaria parasitaemia among symptomatic individuals

Parasite positivity rates, by CareStart™ Malaria HRP2 (Pf), ranged between 21.4% (in the Greater Accra and Upper East regions) and 41.5% in the Brong Ahafo region yielding a national rate of 32.3% whilst parasite positivity rates by microscopy ranged between 8.0% (in the Eastern region) and 23.4% (in the Ashanti region) yielding a national rate of 16.0%. Generally, majority of parasite densities ranged between 10,000 and 99,999 per μL, with the Upper West region showing the highest geometric mean parasite density of 57,739 per μL (Table 2). The proportions of patients with parasite densities less than 200 per μL ranged between 0.0% (in the Eastern and Upper West regions) and 5.1% (in the Central region) yielding a national proportion of 1.8%.

**Table 2:** Malaria parasitaemia by region

Characteristics	Region																					
	Ashanti		B/Ahafo		Central		Eastern		G/Accra		Northern		U/East		U/West		Volta		Western		Overall	
	N	%	N	%	N	%	N	%	N	%	N	%	N	%	N	%	N	%	N	%	N	%
Parasite positivity rates																						
mRDT	2031	37.9	2143	41.5	1446	38.2	1793	36.4	2215	21.4	2186	30.7	1744	21.4	2155	32.2	1698	32.4	1991	32.3	19,402	32.3
Microscopy	1907	23.4	2047	17.0	1377	22.7	1789	8.0	2137	10.3	2123	19.5	1711	10.9	1971	15.9	1648	18.3	1906	15.7	18,616	16.0
Parasite density (per μL)																						
< 200	446	2.2	348	0.3	313	5.1	144	0.0	220	0.5	413	2.7	186	2.7	313	0.0	302	2.6	299	0.7	2984	1.8
200–499	446	4.7	348	1.4	313	4.8	144	0.0	220	1.4	413	3.9	186	1.6	313	0.0	302	3.0	299	2.3	2984	2.6
500–999	446	4.5	348	0.6	313	4.8	144	0.0	220	5.0	413	4.4	186	1.1	313	1.6	302	2.3	299	2.3	2984	2.9
1000–9999	446	23.3	348	18.1	313	29.4	144	18.8	220	30.0	413	21.3	186	15.6	313	9.3	302	22.5	299	20.4	2984	21.0
10,000–99,999	446	45.5	348	48.3	313	44.4	144	47.9	220	55.9	413	26.4	186	39.2	313	48.9	302	54.6	299	56.2	2984	45.9
≥ 100,000	446	19.7	348	31.3	313	11.5	144	33.3	220	7.3	413	41.4	186	39.8	313	40.3	302	14.9	299	18.1	2984	25.7
^a^GMPD (per μL) (min, max)	16,942 (34, 1,096,867)		35,412 (159, 667,142)		10,697 (16, 675,000)		41,744 (1021, 662,500)		13,176 (160, 278,058)		28,856 (48, 965,000)		39,667 (32, 1,037,500)		57,739 (561, 1,625,000)		19,312 (48, 588,000)		22,843 (127, 1,030,000)		24,314 (16, 1,625,000)	
Type of malaria infection																						
Pf	446	99.3	348	99.4	313	99.0	144	97.9	220	99.5	413	97.1	186	99.5	313	97.1	302	92.4	299	99.0	2984	98.0
Pm	446	0.2	348	0.3	313	0.3	144	0.7	220	0.5	413	0.5	186	0.5	313	1.9	302	1.7	299	0.3	2984	0.7
Po	446	0.0	348	0.3	313	0.0	144	0.0	220	0.0	413	0.0	186	0.0	313	1.0	302	1.7	299	0.3	2984	0.3
Pf + Pm	446	0.4	348	0.0	313	0.6	144	0.0	220	0.0	413	2.4	186	0.0	313	0.0	302	1.3	299	0.3	2984	0.6
Pf + Po	446	0.0	348	0.0	313	0.0	144	1.4	220	0.0	413	0.0	186	0.0	313	0.0	302	3.0	299	0.0	2984	0.4
Gametocytaemia	1907	0.4	2047	0.3	1377	0.3	1789	0.0	2137	0.1	2123	0.4	1711	0.2	1971	0.0	1648	0.0	1906	0.2	18,616	0.2

^a^Geometric mean parasite density

*Plasmodium falciparum* (*Pf*) mono-infection was the most prevalent infection type (98.0%), ranging between 92.4% (in the Volta region) and 99.5% (in the Greater Accra and Upper East regions). Prevalence of *Plasmodium malariae* (*Pm*) mono-infection ranged between 0.2% (in the Ashanti region) and 1.9% (in the Upper West region) yielding a national prevalence of 0.7%. *Plasmodium ovale* (*Po*) mono-infection occurred in four regions with prevalence ranging between 0.3% (in the Brong Ahafo region) and 1.7% in the Volta region. Regions with mixed infection prevalence of over 1.0% were Northern (2.4%) and Volta (1.3%) for *Pf + Pm* infection type and Eastern (1.4%) and Volta (3.0%) for *Pf + Po* infection type. There were no *Plasmodium vivax* (*Pv*) infections (Table 2). Generally, prevalence of gametocytaemia was less than 2.0%.

The overall malaria parasite positivity rates by SD Bioline Malaria Ag Pf (HRP2/pLDH) were 31.2% (95% CI: 30.6–31.9) for HRP2 and 25.3% (95% CI: 24.7–25.9) for pLDH.

### Factors associated with malaria parasitaemia among symptomatic individuals

Univariate analysis showed that generally, malaria parasitaemia by microscopy and CareStart™ Malaria HRP2 (Pf) was associated with gender, age, education, and marital status. Malaria parasitaemia by microscopy was further associated with history of antimalarial intake 2 weeks preceding survey (Table 3).

**Table 3:** Univariate analysis for malaria parasitaemia by microscopy and CareStart™ Malaria HRP2 (Pf) in health care facilities in Ghana

Characteristics	Microscopy			CareStart™ Malaria HRP2 (Pf)		
	Total	%	*P*-value	Total	%	*P*-value
Gender						
Male	6131	20.6		6531	39.8	
Female	11,331	14.2	< 0.001	11,945	28.7	< 0.001
Age group (yrs)						
< 5	2907	22.8		3083	39.1	
5–9	2601	31.9		2849	54.6	
10–14	1542	28.5		1651	53.8	
15–19	1567	18.4		1659	38.6	
≥ 20	8845	7.4	< 0.001	9234	18.9	< 0.001
^a^Education						
None	3455	10.6		3617	24.1	
Primary	4288	26.2		2239	48.4	
Junior High	2885	12.8		3026	28.9	
Senior High	2392	10.1		2517	23.4	
Tertiary	1535	7.0	< 0.001	1609	15.7	< 0.001
^b^Marital status						
Single	3587	10.3		3988	26.4	
^c^Married	6084	7.8		6372	19.3	
^d^Divorced	515	7.2	< 0.001	533	19.3	< 0.001
^e^PAMI						
No	15,660	16.9		16,567	32.6	
Yes	1802	12.5	< 0.001	1909	32.8	0.847

^a^Controlled for patients aged < 5 yrs

^b^Controlled for patients aged < 15 yrs

^c^Includes cohabiting

^d^Includes separated

^e^Previous antimalarial intake

Multivariable GEE analysis, which accounted for clustering and controlled for patients aged < 15 years, showed that malaria parasitaemia by microscopy was significantly associated with gender, age, and previous antimalarial intake whilst parasitaemia by CareStart™ Malaria HRP2 (Pf) was significantly associated with gender, age, and education. The odds of parasitaemia by microscopy was significantly lower among female patients compared with males (OR = 0.78; 95% CI: 0.66–0.91); significantly lower among patients aged ≥20 years compared with those aged 15–19 years (OR = 0.37; 95% CI: 0.29–0.46); and significantly lower among patients with history of previous antimalarial intake prior to the survey compared with those with no such history (OR = 0.72; 95% CI: 0.54–0.95) (Table 4).

**Table 4:** Multivariable GEE analysis for malaria parasitaemia by microscopy and CareStart™ Malaria HRP2 (Pf) in health care facilities in Ghana

Characteristics	Microscopy			CareStart™ Malaria HRP2 (Pf)		
	OR	95% CI	*p*-value	OR	95% CI	*p*-value
Gender						
Male^d^						
Female	0.78	0.66–0.91	0.002	0.70	0.62–0.80	< 0.001
Age group (yrs)						
15 – 19^d^						
≥ 20	0.37	0.29–0.46	< 0.001	0.42	0.36–0.50	< 0.001
Education						
None^d^						
Primary	1.10	0.84–1.45	0.478	1.08	0.91–1.29	0.357
Junior High	1.00	0.83–1.19	0.954	1.01	0.90–1.15	0.841
Senior High	0.92	0.74–1.14	0.432	0.88	0.77–1.00	0.056
Tertiary	0.92	0.73–1.15	0.449	0.78	0.65–0.92	0.004
Marital status						
Single^d^						
^a^Married	1.07	0.89–1.30	0457	0.97	0.86–1.09	0.613
^b^Divorced	1.02	0.67–1.55	0.928	0.92	0.73–1.16	0.467
^c^PAMI						
No^d^						
Yes	0.72	0.54–0.95	0.020	0.95	0.79–1.15	0.603

^a^Includes cohabiting

^b^Includes separated

^c^Previous antimalarial intake

^d^Reference category

Similarly, the odds of parasitaemia by CareStart™ Malaria HRP2 (Pf) was significantly lower among females compared with males (OR = 0.70; 95% CI: 0.62–0.80); significantly lower among patients aged ≥20 years compared with those aged 15–19 years (OR = 0.42; 95% CI: 0.36–0.50); and significantly lower among patients with tertiary education compared with those with no formal education (OR = 0.78; 95% CI: 0.65–0.92) (Table 4).

### Diagnostic accuracy of mRDT using microscopy as gold standard

**Fig. 3:**
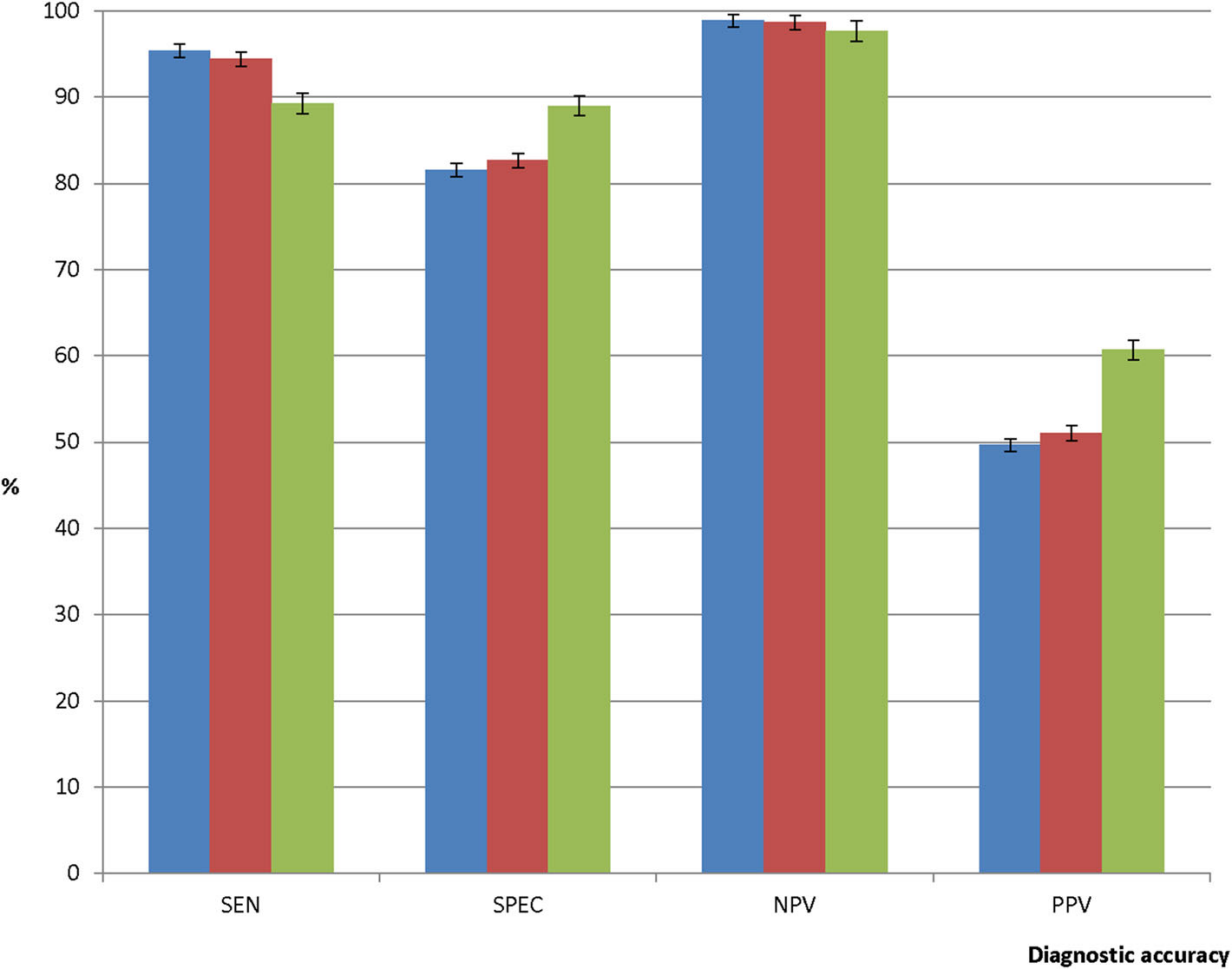
Diagnostic accuracy by mRDT type. Legend details: Blue bar represents CareStart™ Malaria HRP2 (Pf) test kit whilst red and green bars represent HRP2 band and pLDH band, respectively, of SD Bioline Malaria Ag Pf (HRP2/pLDH) combo kit. SEN: Sensitivity; SPEC: Specificity; NPV: Negative predictive value; PPV: Positive predictive value

Using microscopy as gold standard, the overall sensitivity of CareStart™ Malaria HRP2 (Pf) was statistically similar to that of HRP2 band of SD Bioline Malaria Ag Pf (HRP2/pLDH) combo kit (95.4%; 95% CI: 94.6–96.1 vs 94.4%; 95% CI: 93.5–95.2; *p* = 0.093) but significantly higher than pLDH band (95.4%; 95% CI: 94.6–96.1 vs 89.3%; 95% CI: 88.1–90.4; *p* < 0.001). The pLDH band of the SD Bioline Malaria Ag Pf (HRP2/pLDH) combo kit showed the highest specificity compared with the HRP2 band (89.0%; 95% CI: 88.5–89.5 vs 82.7%; 95% CI: 82.1–83.3; *p* < 0.001) and CareStart™ Malaria HRP2 (Pf) (89.0%; 95% CI: 88.5–89.5 vs 81.6%; 95% CI: 81.0–82.2; *p* < 0.001) (Fig. 3). The negative predictive value for the pLDH band of SD Bioline Malaria Ag Pf (HRP2/pLDH) combo kit was significantly lower than the HRP2 band (97.7%; 95% CI: 97.4–97.9 vs 98.7%; 95% CI: 98.5–98.9; *p* < 0.001) and CareStart™ Malaria HRP2 (Pf) (97.7%; 95% CI: 97.4–97.9 vs 98.9%; 95% CI: 98.7–99.1; *p* < 0.001). On the contrary, positive predictive value for the pLDH band of SD Bioline Malaria Ag Pf (HRP2/pLDH) combo kit was significantly the highest compared with the HRP2 band and CareStart™ Malaria HRP2 (Pf) (Fig. 3). The higher sensitivity of the HRP2-based test kits and higher specificity of the pLDH-based test kit was observed in all the regions of Ghana (Additional file 2).

## Discussions

Detection of malaria parasitaemia among symptomatic individuals is key in the provision of effective disease management and surveillance [4]. This study, which was part of a national survey of malaria parasites with HRP2 gene deletions, has shown that Ghana is still in the control phase of the malaria control to elimination continuum [5] with malaria slide positivity rates ranging between 8.0 and 23.4% with a national average of 16.0%. This compares well with the 15.7% slide positivity rate reported from 31 malaria sentinel sites across Ghana in the same year [18]. *Plasmodium falciparum* mono-infection accounted for 98.0% of all infection types, comparable with the 96.3% reported from the national malaria sentinel sites [18].

Malaria parasitaemia by either microscopy or CareStart™ Malaria HRP2 (Pf) among symptomatic individuals reporting at health care facilities was found to be significantly associated with gender and age. Parasitaemia by microscopy was further associated with reported history of antimalarial intake prior to visit to the health care facility whilst parasitaemia by CareStart™ Malaria HRP2 (Pf) was further associated with patient’s educational level.

Female patients were less likely to be parasitaemic either by microscopy (22.0%) or CareStart™ Malaria HRP2 (Pf) (30.0%) compared with males. This finding suggest that women benefit from malaria interventions targeted at children under 5 years by virtue of the fact that they are the primary caretakers of this vulnerable group in most endemic countries [19]. This study brings to the fore the importance of considering males in the deployment of malaria interventions to ensure universal protection. Patients aged ≥20 years were less likely to be parasitaemic either by microscopy (63.0%) or CareStart™ Malaria HRP2 (Pf) (58.0%) compared with those aged 15-19 years, suggesting that patients younger than 20 years should play an important role in reducing prevalence of malaria parasitaemia.

Patients who had a history of previous antimalarial intake during the two-week period before the survey were about 28.0% less likely to be parasitaemic by microscopy compared with those with no previous history of antimalarial intake. This finding compares well with a previous study in the northern region of the country, where children with a reported history of antimalarial intake were over 50.0% less likely to have a positive microscopy result [20]. History of antimalarial intake during the two-week period preceding a suspected malaria patient’s visit to the health care facility can therefore be a good predictor of malaria slide negativity in such patients since the use of antimalarials primarily achieves parasitological cure in addition to clinical cure [3].

The risk of parasitaemia by mRDT, which is a measure of exposure and not only acute infections, was significantly lower among patients with tertiary educational background compared with those with no formal education. Generally, the attainment of higher educational levels translates into higher knowledge levels which affect behavior modification and subsequently incidence and prevalence of malaria and other health events [21, 22]. Promotion of high levels of education, particularly tertiary, should go a long way to reduce exposure to malaria parasites.

With regards to diagnostic accuracy using microscopy as standard, CareStart™ Malaria HRP2 (Pf) kit showed over 90.0% SEN and NPV in all the regions of the country, which compares well with previous studies showing 95.1–98.2% SEN and 95.8–97.7% NPV in parts of the country [23, 24]. Since mRDT is mainly used for diagnosis and not screening, the high SEN and NPV observed in this study suggests that CareStart™ Malaria HRP2 (Pf) kit used routinely in the country is playing a significant role in the proper management of suspected uncomplicated malaria cases [25, 26].

## Conclusions

Ghana remains in the control phase of the malaria control to elimination continuum, with over 90.0% of disease across the country caused by *Plasmodium falciparum*. To facilitate Ghana’s progress from control to pre-elimination phase, the general population should benefit from malaria control interventions. Uptake of these interventions will be enhanced by the promotion of higher level education, particularly tertiary. History of antimalarial intake within the two-week period preceding visit to the clinic is a key predictor of malaria slide negativity, and should therefore guide management of symptomatic individuals. Furthermore, HRP2-based mRDTs remain effective diagnostic tool in the management of suspected uncomplicated malaria in the country.

## Supplementary Information

**Additional file 1.** Questionnaire

**Additional file 2.** mRDT diagnostic accuracy by type and region.

## Data Availability

Data supporting the conclusions made have been included in the article. The dataset for this study will be made available upon reasonable request to the corresponding author.

## Abbreviations

DHIMS: District Health Information Management System
FN: False negative
FP: False positive
HRP2: Histidine-rich protein 2
IPTp: Intermittent preventive treatment in pregnancy
IRS: Indoor residual spraying
LLINs: Long lasting insecticidal nets
mRDT: Malaria rapid diagnostic test
NMCP: National Malaria Control Programme
NMIMR: Noguchi Memorial Institute for Medical Research
NPV: Negative predictive value
OPD: Out-patient department
OR: Odds ratio
*Pf*: *Plasmodium falciparum*
*Pm*: *Plasmodium malariae*
*Po*: *Plasmodium ovale*
pLDH: Parasite lactate dehydrogenase
PPS: Probability proportional to size
PPV: Positive predictive value
*Pv*: *Plasmodium vivax*
QC: Quality control
RBCs: Red blood cells
SEN: Sensitivity
SMC: Seasonal malaria chemoprevention
TN: True negative
TP: True positive
WBCs: White blood cells
WHO: World Health Organization

## References

[1] WHO (2019). World Malaria Report 2019.

[2] Ghana Health Service (2019). National Malaria Control Programme 2018 annual report..

[3] WHO (2015). Guidelines for the treatment of Malaria.

[4] WHO (2000). New perspectives. Malaria diagnosis. Report of a joint WHO/USAID informal consultation. 25-27 October 1999.

[5] WHO (2017). A framework for malaria elimination.

[6] Wirth DF, Casamitjana N, Tanner M, Reich MR (2018). Global action for training in malaria elimination. Malar J.

[7] Moonasar D, Morris N, Kleinschmidt I, Maharaj R, Raman J, Mayet NT (2013). What will move malaria control to elimination in South Africa?. S Afr Med J.

[8] Kateera F, Mens PF, Hakizimana E, Ingabire CM, Muragijemariya L, Karinda P (2015). Malaria parasite carriage and risk determinants in a rural population: a malariometric survey in Rwanda. Malar J.

[9] Adigun AB, Gajere EN, Oresanya O, Vounatsou P (2015). Malaria risk in Nigeria: Bayesian geostatistical modelling of 2010 malaria indicator survey data. Mal J..

[10] Vajda EA, Webb CE (2017). Assessing the risk factors associated with malaria in the highlands of Ethiopia: what do we need to know?. Trop Med Infect Dis.

[11] Steps in applying Probability Proportional to Size (PPS) and calculating Basic Probability Weights. https://who.int/tb/advisory_bodies/impact_measurement_taskforce/meetings/prevalence_survey/psws_probability_prop_size_bierrenbach.pdf?ua=1. Accesed 14 December 2017.

[12] WHO (2018). Protocol for estimating the prevalence of *pfhrp2/pfhrp3* gene deletions among symptomatic falciparum patients with false-negative RDT results.

[13] WHO. Malaria parasite counting. Malaria microscopy standard operating procedure-MM-SOP-09. https://apps.who.int/iris/bitstream/handle/10665/274382/MM-SOP-09-eng.pdf?sequence=14&isAllowed=y

[14] Adu-Gyamfi S, Donkoh WJ, Addo AA (2016). Educational reforms in Ghana: past and present. J Educ Hum Dev.

[15] Ghana, Act of Parliament of the Republic of Ghana Entitled The Children’s Act, 1998. www.ghanaprisons.gov.gh/pdf/Ghana_Childrens_Act-1.pdf. Accessed 31 Dec 2020.

[16] Kalema G, Molenberghs G, Kassahun W (2016). Second-order generalized estimating equations for correlated count data. Comput Stat.

[17] Agresti A (2002). Categorical data analysis.

[18] Abuaku B (2019). Update: Tracking malaria disease prevalence in Ghana.

[19] The Gobal Fund. Technical Brief. Malaria, Gender and Human Rights. https://www.theglobalfund.org/media/5536/core_malariagenderhumanrights_technicalbrief_en.pdf. Accessed 31 December 2020.

[20] Abuaku B, Ahorlu C, Psychas P, Ricks P, Oppong S, Mensah S (2018). Impact of indoor residual spraying on malaria parasitaemia in the Bunkpurugu-Yunyoo district in northern Ghana. Parasit Vectors.

[21] Dike N, Onwujekwe O, Ojukwu J, Ikeme A, Uzochukwu B, Shu E (2006). Influence of education and knowledge on perception and practices to control malaria in Southeast Nigeria. Soc Sci Med.

[22] Diaz-Quijano FA, Martinez-Vega RA, Rodriguez-Morales AJ, Rojas-Calero RA, Luna-Gonzalez ML, Diaz-Quijano RG (2018). Association between the level of education and knowledge, attitudes and practices regarding dengue in the Caribbean region of Colombia. BMC Public Health.

[23] Adu-Gyasi D, Asante KP, Amoako S, Amoako N, Ankrah L, Dosoo D (2015). Assessing the performance of only HRP2 and HRP2 with pLDH based rapid diagnostic tests for the diagnosis of malaria in middle Ghana, Africa. PLoS One.

[24] Quakyi IA, Adjei GO, Sullivan DJ, Laar A, Stephens JK, Owusu R (2018). Diagnostic capacity, and predictive values of rapid diagnostic tests for accurate diagnosis of Plasmodium falciparum in febrile children in Asante-Akim, Ghana. Mal J.

[25] Mandrekar JN (2010). Simple statistical measures for diagnostic accuracy assessment. J Thorac Oncol.

[26] Gerstman BB. Epidemiology kept simple. An introduction to traditional and modern epidemiology. 2nd ed. New Jersey: Wiley-Liss; 2003.

