# Supplementary material for: Malaria parasitaemia and mRDT diagnostic performances among symptomatic individuals in selected health care facilities across Ghana
Source: BMC Public Health. 2021 Jan 28;21:239. doi: 10.1186/s12889-021-10290-1 (PMC7844948; doi:10.1186/s12889-021-10290-1)
Supplement: Supplementary file 1 — Additional file 1. Questionnaire [file 12889_2021_10290_MOESM1_ESM.docx]

Additional file 1. **QUESTIONNAIRE**

**STUDY TITLE:** Determining whether histidine rich protein gene deletions causing negative HRP2 RDT results among symptomatic patients with confirmed *P. falciparum* malaria have reached a threshold for change in diagnostic strategy

| **var#** | **1.Study Profile** | |  |
| --- | --- | --- | --- |
| 101 | Regional code |  |  |
| 102 | Facility code |  |  |
| 103 | Date of interview (YYYY/MM/DD) |  |  |
| 104 | Start Time (HH:MM) |  |  |
|  | **2. Participant Information** | |  |
| 201 | Participant’s serial Number |  |  |
| 202 | Participant’s ID (101+102+201) |  |  |
| 203 | Gender | □ Male □ Female |  |
| 204 | Age of participant (In completed years) |  |  |
| 205 | Participant’s level of formal education | None 01  Primary 02  Junior High 03  Senior High 04  Tertiary 05 |  |
| 206 | Participant’s marital status? | Single 01  Married 02  Cohabiting 03  Separated 04  Divorced 05 |  |
| 207 | Has participant taken any antimalarial in the past 2 weeks?  If No SKIP to 301 | □ Yes □ No |  |
| 208 | What antimalarial did participant take? | Amodiaquine-Artesunate 01  Artemether-Lumefantrine 02  Fansidar / SP 03  Quinine 04  Other (specify) 05 |  |
|  | **3. mRDT Information** |  |  |
| 301a | CareStart HRP2 Lot Number |  |  |
| 301b | CareStart HRP2 Expiry date (YYYY/MM) |  |  |
| 301c | CareStart HRP2 Results | □ Positive □ Negative |  |
| 301d | Picture of CareStart HRP2 Results uploaded | □ Yes □ No |  |
| 302a | SD Bioline HRP2/pLDH Lot Number |  |  |
| 302b | SD Bioline HRP2/pLDH Expiry date (YYYY/MM) |  |  |
| 302c | SD Bioline HRP2 Results | □ Positive □ Negative |  |
| 302d | SD Bioline pLDH Results | □ Positive □ Negative |  |
| 302e | Picture of SD Bioline HRP2/pLDH Results uploaded | □ Yes □ No |  |
|  | **4. Checklist** |  |  |
| 401 | Blood smears prepared for microscopy | □ Yes □ No |  |
| 402 | Filter paper blots taken | □ Yes □ No |  |
| 403 | End Time (HH:MM) |  |  |
