# Supplementary material for: Malaria parasitaemia and mRDT diagnostic performances among symptomatic individuals in selected health care facilities across Ghana
Source: BMC Public Health. 2021 Jan 28;21:239. doi: 10.1186/s12889-021-10290-1 (PMC7844948; doi:10.1186/s12889-021-10290-1)
Supplement: Supplementary file 2 — Additional file 2. mRDT diagnostic accuracy by type and region. [file 12889_2021_10290_MOESM2_ESM.docx]

Additional file 2. mRDT diagnostic accuracy by type and region

| Region | CareStart HRP2 results | Microscopy results | | Diagnostic accuracy | | | |
| --- | --- | --- | --- | --- | --- | --- | --- |
|  |  | Negative | Positive | SEN (95% CI) | SPEC (95% CI) | NPV (95% CI) | PPV (95% CI) |
| Ashanti  (N=1880) | Negative | 1170 | 26 | 94.0 (91.2-96.0) | 81.0 (78.9-83.0) | 97.8 (96.8-98.5) | 59.8 (56.0-63.5) |
|  | Positive | 275 | 409 |  |  |  |  |
|  |  |  |  |  |  |  |  |
| B/Ahafo  (N=2044) | Negative | 1230 | 9 | 97.4 (95.0-98.7) | 72.4 (70.2-74.5) | 99.3 (98.6-99.7) | 41.9 (38.5-45.4) |
|  | Positive | 468 | 337 |  |  |  |  |
|  |  |  |  |  |  |  |  |
| Central  (N=1370) | Negative | 842 | 26 | 91.7 (87.9-94.4) | 79.6 (77.0-82.0) | 97.0 (95.6-98.0) | 57.0 (52.5-61.4) |
|  | Positive | 216 | 286 |  |  |  |  |
|  |  |  |  |  |  |  |  |
| Eastern  (N=1620) | Negative | 1037 | 6 | 95.5 (90.0-98.2) | 69.7 (67.3-72.0) | 99.4 (98.7-99.8) | 21.8 (18.5-25.4) |
|  | Positive | 451 | 126 |  |  |  |  |
|  |  |  |  |  |  |  |  |
| G/Accra  (N=2115) | Negative | 1683 | 6 | 97.2 (93.8-98.9) | 88.7 (87.2-90.1) | 99.6 (99.1-99.8) | 49.5 (44.7-54.4) |
|  | Positive | 215 | 211 |  |  |  |  |
|  |  |  |  |  |  |  |  |
| Northern  (N=2104) | Negative | 1479 | 16 | 96.1 (93.6-97.7) | 87.2 (85.5-88.7) | 98.9 (98.2-99.3) | 64.2 (60.2-68.0) |
|  | Positive | 218 | 391 |  |  |  |  |
|  |  |  |  |  |  |  |  |
| U/East  (N=1591) | Negative | 1259 | 13 | 92.7 (87.6-95.9) | 89.2 (87.4-90.8) | 99.0 (98.2-99.5) | 52.0 (46.4-57.6) |
|  | Positive | 153 | 166 |  |  |  |  |
|  |  |  |  |  |  |  |  |
| U/West  (N=1962) | Negative | 1365 | 20 | 93.4 (89.8-95.8) | 82.3 (80.4-84.1) | 98.6 (97.8-99.1) | 49.2 (45.1-53.4) |
|  | Positive | 293 | 284 |  |  |  |  |
|  |  |  |  |  |  |  |  |
| Volta  (N=1632) | Negative | 1126 | 4 | 98.6 (96.3-99.5) | 84.0 (81.9-85.9) | 99.6 (99.5-99.7) | 57.4 (52.9-61.8) |
|  | Positive | 214 | 288 |  |  |  |  |
|  |  |  |  |  |  |  |  |
| Western  (N=1902) | Negative | 1286 | 8 | 97.3 (94.6-98.7) | 80.1 (78.0-82.0) | 99.2 (98.8-99.7) | 47.5 (43.5-51.6) |
|  | Positive | 319 | 289 |  |  |  |  |
|  |  |  |  |  |  |  |  |
| Overall  (N=18220) | Negative | 12477 | 134 | 95.4 (94.6-96.1) | 81.6 (81.0-82.2) | 98.9 (98.7-99.1) | 49.7 (48.4-51.0) |
|  | Positive | 2822 | 2787 |  |  |  |  |

| Region | SD Bioline HRP2 results | Microscopy results | | Diagnostic accuracy | | | |
| --- | --- | --- | --- | --- | --- | --- | --- |
|  |  | Negative | Positive | SEN (95% CI) | SPEC (95% CI) | NPV (95% CI) | PPV (95% CI) |
| Ashanti  (N=1880) | Negative | 1203 | 30 | 93.1 (90.2-95.2) | 83.3 (81.3-85.2) | 97.6 (96.5-98.4) | 62.6 (58.7-66.3) |
|  | Positive | 242 | 405 |  |  |  |  |
|  |  |  |  |  |  |  |  |
| B/Ahafo  (N=2041) | Negative | 1241 | 10 | 97.1 (94.6-98.5) | 73.2 (71.0-75.3) | 99.2 (98.5-99.6) | 42.5(39.0-46.0) |
|  | Positive | 454 | 336 |  |  |  |  |
|  |  |  |  |  |  |  |  |
| Central  (N=1350) | Negative | 839 | 31 | 90.0 (86.0-93.0) | 80.7 (78.1-83.0) | 96.4 (94.9-97.5) | 58.1 (53.5-62.5) |
|  | Positive | 201 | 279 |  |  |  |  |
|  |  |  |  |  |  |  |  |
| Eastern  (N=1620) | Negative | 1037 | 6 | 95.5 (90.0-98.2) | 71.2 (68.8-73.5) | 99.4 (98.7-99.8) | 22.7 (19.3-26.5) |
|  | Positive | 451 | 126 |  |  |  |  |
|  |  |  |  |  |  |  |  |
| G/Accra  (N=2112) | Negative | 1687 | 6 | 97.2 (93.7-98.9) | 89.0 (87.5-90.4) | 99.6 (99.1-99.8) | 50.1 (45.2-55.0) |
|  | Positive | 209 | 210 |  |  |  |  |
|  |  |  |  |  |  |  |  |
| Northern  (N=2106) | Negative | 1513 | 32 | 92.2 (89.1-94.5) | 89.1 (87.5-90.5) | 97.9 (97.0-98.5) | 67.0 (62.9-70.9) |
|  | Positive | 185 | 376 |  |  |  |  |
|  |  |  |  |  |  |  |  |
| U/East  (N=1696) | Negative | 1362 | 18 | 90.2 (84.7-93.9) | 90.1 (88.5-91.5) | 98.7 (97.9-99.2) | 52.5 (49.5-60.7) |
|  | Positive | 150 | 166 |  |  |  |  |
|  |  |  |  |  |  |  |  |
| U/West  (N=1962) | Negative | 1373 | 19 | 93.8 (90.3-96.1) | 82.8 (80.9-84.6) | 98.6 (97.8-99.1) | 50.0 (45.8-54.2) |
|  | Positive | 285 | 285 |  |  |  |  |
|  |  |  |  |  |  |  |  |
| Volta  (N=1637) | Negative | 1133 | 5 | 98.3 (95.8-99.4) | 84.2 (82.1-86.1) | 99.6 (99.0-99.9) | 57.5 (53.0-61.9) |
|  | Positive | 212 | 287 |  |  |  |  |
|  |  |  |  |  |  |  |  |
| Western  (N=1685) | Negative | 1145 | 5 | 98.2 (95.6-99.3) | 81.4 (79.3-83.4) | 99.6 (99.0-99.7) | 51.0 (46.7-55.3) |
|  | Positive | 262 | 273 |  |  |  |  |
|  |  |  |  |  |  |  |  |
| Overall  (N=18089) | Negative | 12555 | 162 | 94.4 (93.5-95.2) | 82.7 (82.1-83.3) | 98.7 (98.5-98.9) | 51.1 (49.8-52.4) |
|  | Positive | 2629 | 2743 |  |  |  |  |

| Region | SD Bioline pLDH results | Microscopy results | | Diagnostic accuracy | | | |
| --- | --- | --- | --- | --- | --- | --- | --- |
|  |  | Negative | Positive | SEN (95% CI) | SPEC (95% CI) | NPV (95% CI) | PPV (95% CI) |
| Ashanti  (N=1880) | Negative | 1263 | 61 | 86.0 (82.3-89.1) | 87.4 (85.6-89.0) | 95.4 (94.1-96.4) | 67.3 (63.2-71.2) |
|  | Positive | 182 | 374 |  |  |  |  |
|  |  |  |  |  |  |  |  |
| B/Ahafo  (N=2041) | Negative | 1399 | 23 | 93.4 (90.1-95.7) | 82.5 (80.6-84.3) | 98.4 (97.6-99.0) | 52.2 (48.2-56.2) |
|  | Positive | 296 | 323 |  |  |  |  |
|  |  |  |  |  |  |  |  |
| Central  (N=1350) | Negative | 936 | 52 | 83.2 (78.5-87.1) | 90.0 (88.0-91.7) | 94.7 (93.1-96.0) | 71.3 (66.3-75.9) |
|  | Positive | 104 | 258 |  |  |  |  |
|  |  |  |  |  |  |  |  |
| Eastern  (N=1620) | Negative | 1175 | 17 | 87.1 (79.9-92.1) | 79.0 (76.8-81.0) | 98.6 (97.7-99.2) | 26.9 (22.8-31.4) |
|  | Positive | 313 | 115 |  |  |  |  |
|  |  |  |  |  |  |  |  |
| G/Accra  (N=2112) | Negative | 1721 | 8 | 96.3 (92.6-98.3) | 90.8 (89.4-92.0) | 99.5 (99.0-99.8) | 54.3 (49.2-59.4) |
|  | Positive | 175 | 208 |  |  |  |  |
|  |  |  |  |  |  |  |  |
| Northern  (N=2106) | Negative | 1591 | 53 | 87.0 (83.3-90.0) | 93.7 (92.4-94.8) | 96.8 (95.8-97.6) | 76.8 (72.6-80.5) |
|  | Positive | 107 | 355 |  |  |  |  |
|  |  |  |  |  |  |  |  |
| U/East  (N=1696) | Negative | 1395 | 28 | 84.8 (78.6-89.5) | 92.3 (90.8-93.6) | 98.0 (97.1-98.6) | 57.1 (51.0-63.0) |
|  | Positive | 117 | 156 |  |  |  |  |
|  |  |  |  |  |  |  |  |
| U/West  (N=1962) | Negative | 1522 | 38 | 87.5 (83.1-90.9) | 91.8 (90.4-93.1) | 97.6 (96.7-98.3) | 66.2 (61.3-70.8) |
|  | Positive | 136 | 266 |  |  |  |  |
|  |  |  |  |  |  |  |  |
| Volta  (N=1637) | Negative | 1235 | 11 | 96.2 (93.1-98.0) | 91.8 (90.2-93.2) | 99.1 (98.4-99.5) | 71.9 (67.1-76.3) |
|  | Positive | 110 | 281 |  |  |  |  |
|  |  |  |  |  |  |  |  |
| Western  (N=1685) | Negative | 1270 | 20 | 92.8 (88.9-95.4) | 90.3 (88.6-91.8) | 98.4 (97.5-99.0) | 65.3 (60.4-70.0) |
|  | Positive | 137 | 258 |  |  |  |  |
|  |  |  |  |  |  |  |  |
| Overall  (N=18089) | Negative | 13507 | 311 | 89.3 (88.1-90.4) | 89.0 (88.5-89.5) | 97.7 (97.4-97.9) | 60.7 (59.2-62.2) |
|  | Positive | 1677 | 2594 |  |  |  |  |
